# Supplementary material for: Immunological memory in a teleost fish: common carp IgM+ B cells differentiate into memory and plasma cells
Source: Front Immunol. 2024 Dec 20;15:1493840. doi: 10.3389/fimmu.2024.1493840 (PMC11695322; doi:10.3389/fimmu.2024.1493840)
Supplement: Supplementary file 2 [file DataSheet2.pdf]

Supplementary Table

Table S1 Summary of all the oligonucleotides designed for this study against various (predicted) B cell or B cell-related markers. Oligonucleotides are divided into two groups: the first five primers target predicted (in common carp) B cell-associated markers whose expression we measured as part of Figure 4 and the Method Details section ‘Quantitative reverse transcription PCR (RT-qPCR) gene expression profiling of B cell activation and differentiation markers’; the second group includes primers targeting 18 *Cyprinus carpio* orthologues of grass carp B cell-associated genes that Pan et al. (1) identified by single-cell RNA sequencing as defining and clustering distinct head kidney IgM$^+$ B cell populations. We measured the latter group of genes as part of Figure 5 and the Method Details section ‘Multiplex qPCR gene expression profiling’. These two groups of primers are divided by a dashed line.

Gene symbol	NCBI or GenBank accession code	Gene product	Forward (F) and reverse (R) primers (5’-3’)	Primer efficiencies (%)	Amplicon length (bp)
tnfrsf13b	XM_042734762.1	*Cyprinus carpio* tumor necrosis factor receptor superfamily member 13B	F: CAGTGCTCCGAGCTGTGT<br>R: AGTACAGCGCTGATCCGGA	98.68	157
xbp1	XM_042753507.1	*Cyprinus carpio* X-box binding protein 1	F: AAAGCACTTCGAAGGAAACTGAAG<br>R: CGAGCTCCAACTCCAAGACTT	97.91	109
syndecan-3-like	XM_042777052.1	*Cyprinus carpio* syndecan-3-like	F: GTCTCTTATGGCCACATTTACTCG<br>R: CCCACAGCAATAATGTCCCAAAA	96.72	122

pax5	XM_019109396.2<br>XM_019109382.2<br>XM_019109370.2<br>XM_019109360.2	*Cyprinus carpio* paired box 5, transcript variants X1, X2, X3, and X4	F: GGCTATGGCGTCTTTGGCT<br>R: TCGCGACCAGACACAAGTG	97.06	123
cxcr5	XM_042743592.1<br>XM_042743591.1	Cyprinus carpio C-X-C chemokine receptor type 5, transcript variants X1 and X2	F: CTTAAACGGCGGAGGAACCT<br>R: GCCCAATAGCTTGCAGAGGA	100.51	150
cd22	XM_042754392, XM_042726754	*Cyprinus carpio* cluster of differentiation 22	F: CATTCTCTTGCTGATGTCCTTCAT<br>R: ATGAGCGTGTGTTCAGAGGAGA	88.48	99
cd34	XM_042713470	*Cyprinus carpio* cluster of differentiation 34	F: CTTCACAGTTGCTGGGGACAG<br>R: AACAGGTTCAAAAGCAGGTCCAA	85.39	109
cd79b	XM_042767177	*Cyprinus carpio* cluster of differentiation 79b	CCTCTCAGCTGAAGTAAATATCC,<br>TGACATCTGATCCATCTTTGACG	92.74	109
cd80	XM_042740109	*Cyprinus carpio* cluster of differentiation 80	CACAGCTGAGTACAGCGTTCC,<br>AGGTGGACGGGGCTTAATCAAA	95.26	143
cd83	XM_019119505	*Cyprinus carpio* cluster of differentiation 83	CCGTCATATGGTACAAGGTTTCT,<br>TGTCTAATGTAACTGCAGAGGAC	87.66	173

cd86	XM_042778525	*Cyprinus carpio* cluster of differentiation 86	GTGCGCCATTCTTCATTAAGGG, CTACATGAGGACGTCAGACTAG	92.14	148
cxcr4a	XM_042725575	*Cyprinus carpio* C-X-C chemokine receptor type 4 alpha	TACGAACACATCGTCTTTGAAGAT, GAGCCAACTTTGAGGTTCCGTG	94.77	92
cxcr4b	XM_042763477	*Cyprinus carpio* C-X-C chemokine receptor type 4 beta	CTTCTTCATCTGTTGGTTGCCTTA, TGCTGTCTCAACCCCATCCTTTA	93.73	165
egr1	XM_019064348	*Cyprinus carpio* early growth response protein1	ACTGGAGACACGCTTTCAGAAAT, TCTTACACGGGCCGTTTCACC	97.00	102
ier2	XM_019067280	*Cyprinus carpio* immediate early response protein 2	CTCTAGAAAGCGACGGAGCAAA, CGTGCCTATGCCAAGAACAATTG	94.13	173
ighm	AB004106, AB004107, MH352354, MH352353	*Cyprinus carpio* immunoglobulin M heavy chain membrane-bound and secretory protein	CGAATATGCAGTTCCTATTCAAGA T AACATGTGGAACTTGATGCCCC	97.55	216
irf4	XM_019126566	*Cyprinus carpio* interferon regulatory factor 4	GGCCCTCTCAGATTACCGCTTA, TAGTCCAGAGGGCTGTCAGCT	103.31	93
irf8	XM_042754085, XM_042743728	*Cyprinus carpio* interferon regulatory factor 8	ATCTTCAAAGCGTGGGCGATATT, ATTTTGAGGAAGTTACTGACCGAT	91.43	130

klf2	XM_019065449	*Cyprinus carpio* Krüppel like factor 2 alpha	GAAAACAGGTGGAAGGAGGAAC, TTATTCTGGCCAACACTGTGGG	86.07	146
mki67	XM_042735109	*Cyprinus carpio* marker of proliferation Ki-67	GTTCGGAAGGAAGCTGGACTG, AGAACAAGGAGCTCATTTTGACC	100.37	103
pax5	XM_019109360	*Cyprinus carpio* paired box 5	CAACAGGATCATTCGCACTAAAG, GTGACCCAGGTATCTGCAGTAA	87.60	107
top2a	XM_042735154	*Cyprinus carpio* DNA topoisomerase II alpha	ACTCAGCAAATGTGGGTGTTTGAT, GGTTAACATTGACTCGGAGAATAA	99.96	170
xbp1	XM_042753507	*Cyprinus carpio* X-box binding protein 1	TTGGAGTTGGAGCTCGAGAATC, GACTGGGGTTAGATACCCTGG	93.73	121

(1) Pan Y, Wu C, Zhong Y, Zhang Y, Zhang X. An Atlas of Grass Carp IgM+ B Cells in Homeostasis and Bacterial Infection Helps to Reveal the Unique Heterogeneity of B Cells in Early Vertebrates*. J Immunol* (2023) **211**:964–980. doi: 10.4049/jimmunol.2300052.
